# P-763. Evaluation of antibiotic prophylaxis for percutaneous nephrostomy tube procedures

**DOI:** 10.1093/ofid/ofaf695.974

**Published:** 2026-01-11

**Authors:** Andrea H Son, Katelyn Malena, Olivia Pauly, Michelle T Hecker

**Affiliations:** The MetroHealth System, Cleveland, Ohio; MetroHealth Medical Center, Cleveland, Ohio; MetroHealth Medical Center, Cleveland, Ohio; The MetroHealth System, Cleveland, Ohio

## Abstract

**Background:**

Antibiotic prophylaxis for percutaneous nephrostomy tube (PCNT) placements or exchanges is recommended for high-risk patients or those with a symptomatic urinary tract infection (UTI). High-risk factors variously cited in the literature include advanced age, diabetes, indwelling urinary catheters, neurogenic bladder, previous percutaneous ureteral procedures/manipulations, calculi, ileal conduit, and catheter occlusion at time of exchange. We sought to evaluate our institution’s current practices.

**Methods:**

Retrospective chart review of adult patients who had a PCNT procedure in the outpatient setting from 10/1/2023 – 9/30/2024. We defined high-risk as age ≥ 65, diabetes, immunocompromised state, presence of foley or suprapubic catheter, ileal conduit, and blocked or dislodged catheter at the time of catheter exchange.

**Results:**

Of 61 patients reviewed , 29 (48% ) were male and the mean age was 61 (range 24 – 90). This was the initial PCNT placement for 17 (28%) patients. The most common indications for PCNT were obstruction due to cancer 22 (36%), stricture 19 (31%), and stones 17 (28%). Forty-five patients (74%) were considered high-risk of whom 37 (82%) received prophylaxis and 8 (18%) did not. Of the 16 patients considered low-risk, 13 (81%) received prophylaxis and 3 (19%) did not. Ceftriaxone (46%) and ciprofloxacin (44%) were most used. The antibiotic given as prophylaxis was not active against the most recent urine culture in 10 of the 50 (20%) patients who received prophylaxis. Within 30 days after the procedure, 20 (33%) patients were treated for a UTI of whom 7 were hospitalized, 2 had an ED visit only, and 11 were treated in outpatient setting only. Fourteen of 50 (28%) patients who received prophylaxis were treated for a UTI compared to 6 of 11 (55%) patients who did not receive prophylaxis.

**Conclusion:**

At our institution most patients, regardless of risk factors, received antibiotic prophylaxis prior to PCNT procedures, although some high-risk patients did not. Antibiotic choices varied and were not always active against organisms isolated in the most recent urine cultures. UTI treatment within 30 days of PCNT procedure was relatively common and more common in those who did not receive prophylaxis.

**Disclosures:**

All Authors: No reported disclosures

